# Assessment of antibodies in the upper and lower human respiratory tract at steady state and after respiratory viral infection

**DOI:** 10.1002/cti2.1460

**Published:** 2023-08-08

**Authors:** Marios Koutsakos, Jackson S Turner, M Cristina Vazquez Guillamet, Daniel Reynolds, Tingting Lei, Derek E Byers, Ali H Ellebedy, Philip A Mudd

**Affiliations:** ^1^ Department of Microbiology and Immunology The University of Melbourne at the Peter Doherty Institute for Infection and Immunity Melbourne VIC Australia; ^2^ Department of Pathology and Immunology Washington University School of Medicine Saint Louis MO USA; ^3^ Division of Pulmonology and Critical Care, Department of Medicine Washington University School of Medicine Saint Louis MO USA; ^4^ Division of Infectious Diseases, Department of Medicine Washington University School of Medicine Saint Louis MO USA; ^5^ Center for Vaccines and Immunity to Microbial Pathogens Washington University School of Medicine Saint Louis MO USA; ^6^ The Andrew M. and Jane M. Bursky Center for Human Immunology & Immunotherapy Programs Washington University School of Medicine Saint Louis MO USA; ^7^ Department of Emergency Medicine Washington University School of Medicine Saint Louis MO USA

**Keywords:** antibodies, BAL, mucosal immunity, nasopharyngeal swab

## Abstract

**Objectives:**

There is an increasing appreciation for the need to study mucosal antibody responses in humans. Our aim was to determine the utility of different types of samples from the human respiratory tract, specifically nasopharyngeal (NP) swabs obtained for diagnostic purposes and bronchoalveolar lavage (BAL) obtained in outpatient and inpatient settings.

**Methods:**

We analysed antibody levels in plasma and NP swabs from 67 individuals with acute influenza as well as plasma and BAL from individuals undergoing bronchoscopy, including five control subjects as well as seven moderately and seven severely ill subjects with a respiratory viral infection. Levels of α2‐macroglobulin were determined in BAL and plasma to assess plasma exudation.

**Results:**

IgG and IgA were readily detectable in BAL and NP swabs, albeit at different ratios, while IgM levels were low. The total amount of antibody recovered from NP swabs varied greatly between study participants. Accordingly, the levels of influenza HA‐specific antibodies varied, and individuals with lower amounts of total Ig in NP swabs had undetectable levels of HA‐specific Ig. Similarly, the total amount of antibody recovered from BAL varied between study participants. However, severely ill patients showed evidence of increased plasma exudation, which may confound analysis of their BAL samples for mucosal antibodies.

**Conclusion:**

Nasopharyngeal swabs collected for diagnostic purposes may have utility in assessing antibodies from the human nasal mucosa, but variability in sampling should be accounted for. BAL samples can be utilised to study antibodies from the lower respiratory tract, but the possibility of plasma exudation should be excluded.

## Graphical abstract

In this study, we have assessed the utility and limitations of different types of samples from the human respiratory tract, specifically nasopharyngeal swabs obtained for diagnostic purposes and bronchoalveolar lavage obtained in outpatient and inpatient settings. Our findings provide important insights for the assessment of antibodies in the upper and lower respiratory tract of humans.

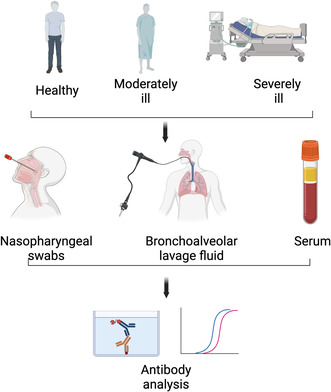

## Introduction

Antibodies are an important component of protective immunity against respiratory viral infections like influenza and SARS‐CoV‐2. For antibodies to provide protection against infection with such pathogens, they must be present and exert their functions at the site of viral entry, the respiratory tract. However, antibody responses to infection or vaccination are typically assessed in serum or plasma from circulating blood. Although antibodies in the circulation can serve as correlates of protection,^1^ understanding the generation and maintenance of mucosal antibody responses in humans remains largely unknown.

Because of the SARS‐CoV‐2 pandemic, there has been increasing interest in measuring antibodies in the human respiratory tract before, during or after infection and/or vaccination. Such measurements could provide critical insights into the induction of mucosal immunity by different vaccination regimes, the longevity of mucosal immunity and ultimately correlates of protection from infection and severe disease. Attempts to study mucosal antibodies in such contexts have been primarily focused on the analyses of saliva samples (recently reviewed^2^). However, the extent to which saliva recapitulates antibodies in the nasal mucosa remains unknown and saliva may not reflect antibodies in the lower respiratory tract. Immunity in the nasal mucosa can be assayed in nasal wash samples^3^, ^4^; such procedures, however, are logistically challenging. While a minority of studies have assessed nasopharyngeal swabs for the presence of antibodies,^5^, ^6^, ^7^, ^8^ their utility in assessing upper respiratory tract immunity remains unclear. Similarly, immunity in the lower respiratory tract may be assessed in bronchoalveolar lavage (BAL) samples, which are typically collected as part of bronchoscopy procedures on severely ill patients. In the context of such severe respiratory infections, it is important to consider whether the underlying tissue damage and excessive plasma exudation confound the analysis of mucosal antibodies in such samples. To address these questions, we analysed antibody levels in NP swabs and BAL samples, as well as paired plasma, from different cohorts.

## Results

### Detection of antibodies in BAL samples and nasopharyngeal swabs from healthy subjects

**Figure 1:**
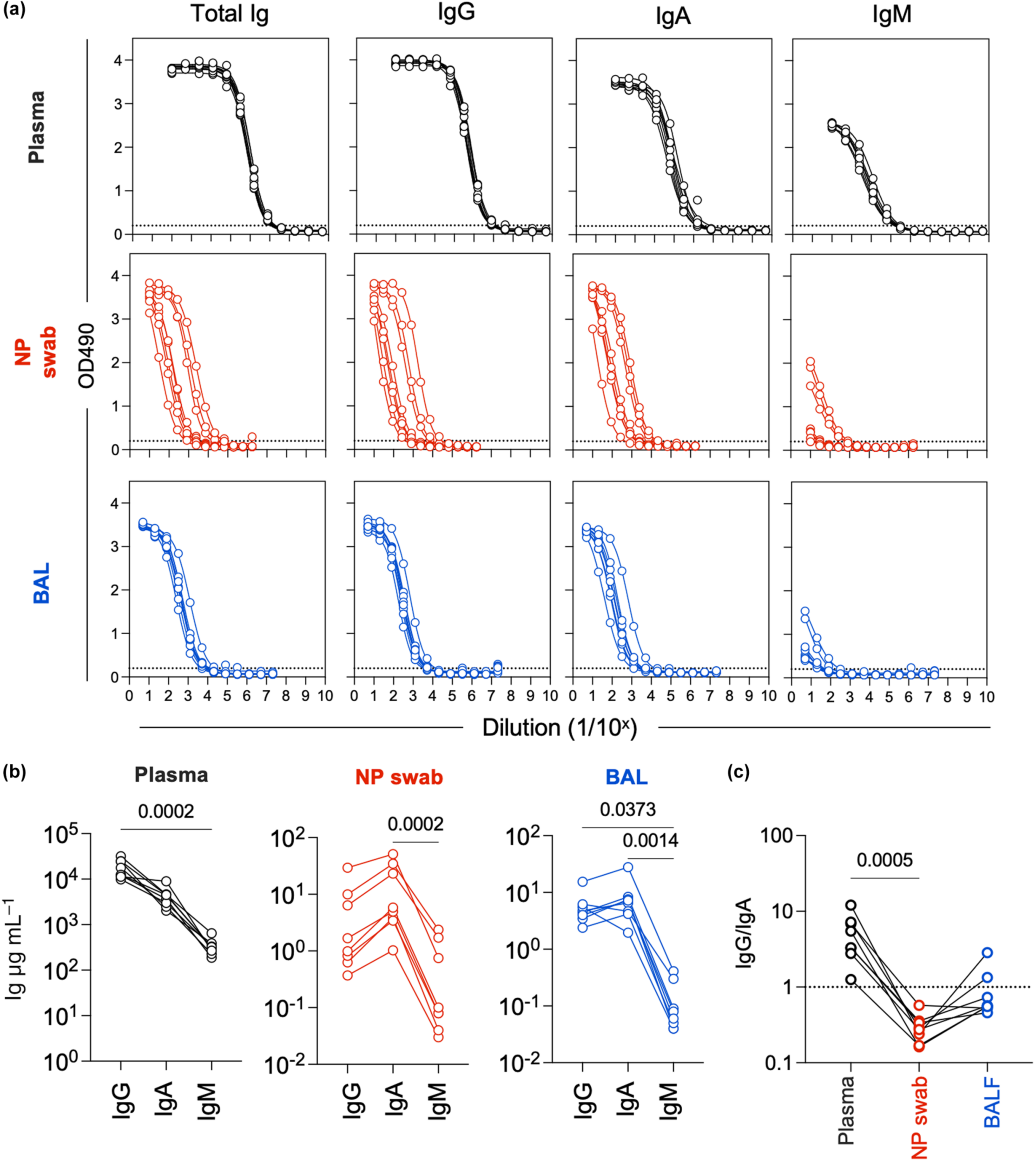
Detection of antibodies in BAL samples and nasopharyngeal swabs. **(a)** ELISA curves from plasma, NP swab and BAL for total Ig (heavy and light chain), IgG, IgA and IgM. Measured optical density at 490 nm and a fitted sigmoidal 4 parameter logistic curves are shown. Each curve represents a different subject. The dotted horizontal line represents the cut‐off value for endpoint titre calculations (3× background signal). **(b)** Interpolated concentrations of IgG, IgA and IgM for paired plasma, NP swab and BAL samples. **(c)** Ratios of IgG and IgA concentrations in paired samples. Samples from control subjects were used for this figure (*n* = 8). Statistical significance was determined by a Friedman's test with Dunn's correction for multiple comparisons.

We first assessed the presence of antibodies of different isotypes in eight healthy subjects undergoing elective outpatient research bronchoscopy. Paired plasma, NP swabs and BAL samples were assessed for the presence of antibodies regardless of isotype (Figure 1a) as well as endpoint titres of IgG, IgA or IgM antibodies (Figure 1a). IgG and IgA antibodies were readily detectable in all sample types and across all donors, while IgM was barely detectable in all samples (Figure 1a and Supplementary figure 1a). In a complementary approach, we used standard curves (Supplementary figure 1b) to determine the concertation (μg mL^−1^) of each isotype in different samples. Interpolated concentrations for the different isotypes were well correlated with matched endpoint titres (Spearman *r* = 0.99, *P* < 0.001) (Supplementary figure 1c). Although the use of different secondary antibodies to detect different isotypes may confound their comparison, we note that the interpolated concentrations of all isotypes from plasma are in accordance with reference values for each isotype^9^ and the interpolated concentrations from BAL are in accordance with previous studies.^10^ At the very least, our data clearly demonstrate that both IgG and IgA are present in samples from both the upper and lower respiratory tract but at different ratios (Figure 1c).

### Variability in sampling of antibodies using NP swabs

**Figure 2:**
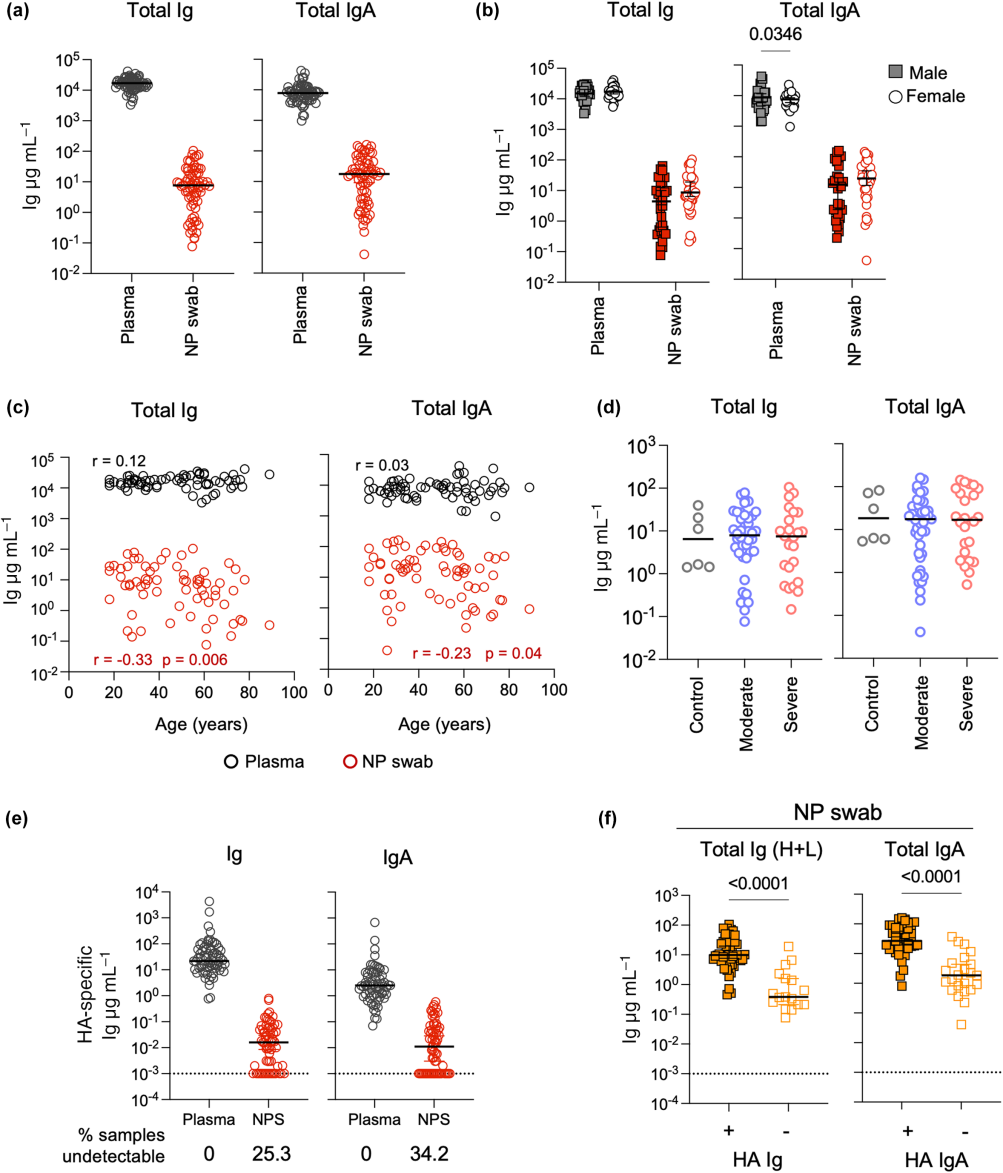
Variability in sampling of antibodies from nasopharyngeal swabs. **(a)** Concentrations of total Ig and IgA in plasma and NP swabs from EDFLU subjects (*n* = 67). Each datapoint represents a different subject and the line shows the median. **(b)** Concentrations of total Ig and IgA in plasma and NP swabs from EDFLU subjects grouped by sex (male *n* = 30, female *n* = 37). Median and 95% confidence intervals are shown. Statistical significance was determined by a two‐way ANOVA with Sidak's correction for multiple comparisons. **(c)** Correlation between concentrations of total Ig and IgA in plasma or NP swabs and age. Spearman's *r* coefficient and *P*‐value are shown for statistically significant comparisons. **(d)** Concentrations of total Ig and IgA in NP swabs from EDFLU subjects grouped based on disease severity (moderate *n* = 43, severe *n* = 24) and control subjects (*n* = 6). Statistical significance was assessed by a Kruskal–Wallis test with Dunn's correction for multiple comparisons. **(e)** Concentrations of HA‐specific Ig and IgA in plasma and NP swabs from EDFLU subjects (*n* = 67). Dotted horizontal line represents the limit of detection. Samples with undetectable levels of HA‐specific antibodies were imputed as 0.001 μg mL^−1^. **(f)** Concentrations of total Ig and IgA in plasma and NP swabs from EDFLU subjects grouped based on whether HA‐specific antibodies were detectable (*n* = 50 for Ig, *n* = 44 for IgA) or not (*n* = 17 for Ig, *n* = 23 for IgA). Statistical significance was assessed by a Mann–Whitney test.

In our analysis of antibody levels from NP swabs (Figure 1), we noted a considerable degree of variability in the total amounts of IgG and IgA recovered for each subject. To further characterise this variability, we assessed NP swabs collected as part of the EDFLU study^11^ from 67 subjects presenting to the Emergency Department with acute influenza. These NP swabs were collected in addition to diagnostic swabs and stored for further analyses. We quantified the amount of antibody in these NP swabs and found extensive variability across the 67 subjects, ranging from 0.08 to 105.3 μg mL^−1^ of total antibody (coefficient of variation 137.3%) (Figure 2a). The levels of IgA varied similarly (coefficient of variation 124.8%). This contrasted with the more homogenous levels of total and IgA antibodies in plasma from the same subjects (coefficient of variation 43.2% and 79.4% respectively). The amount of antibody recovered from NP swabs was not associated with the sex of the subject (Figure 2b) and was only modestly affected by age (Spearman *r* = −0.33, *P* = 0.006 for total Ig; Spearman *r* = −0.23, *P* = 0.04 for IgA) (Figure 2c). Plasma levels of total Ig or IgA were not correlated with age. The total amounts of Ig and IgA recovered from NP swabs or plasma were not different when subjects were grouped as moderately ill (*n* = 43) or severely ill subjects (*n* = 24) (median sampling time of 3 and 4 days post symptom onset respectively) and compared to NP swabs from control subjects (*n* = 6) (Figure 2d, Supplementary figure 1d).

The substantial variability observed needs to be considered in the analysis of antigen‐specific antibody titres from NP swabs. Indeed, when we assessed the antibody tires against influenza virus HA in this cohort (matched to the infecting subtype), 25.3% (17/67) and 34.2% (23/67) had undetectable levels of total HA‐specific and IgA HA‐specific antibodies, respectively, in their NP swabs, despite all plasma samples having detectable levels (Figure 2e). Importantly, NP swabs for which HA‐specific antibodies were undetectable had significantly lower overall antibody levels recovered (Figure 2f). Consistently, the levels HA‐specific antibodies detected in NP swabs were correlated to the total amount of Ig detected (Supplementary figure 1e). This result highlights how variability in sampling may affect the detection of antigen‐specific antibodies. It would therefore be important to normalise antigen‐specific antibody measurements to the total amount of antibody recovered to account for the extensive variability in sampling.

### Increased plasma exudation in BAL samples from critically ill patients may confound the assessment of mucosal antibodies

**Figure 3:**
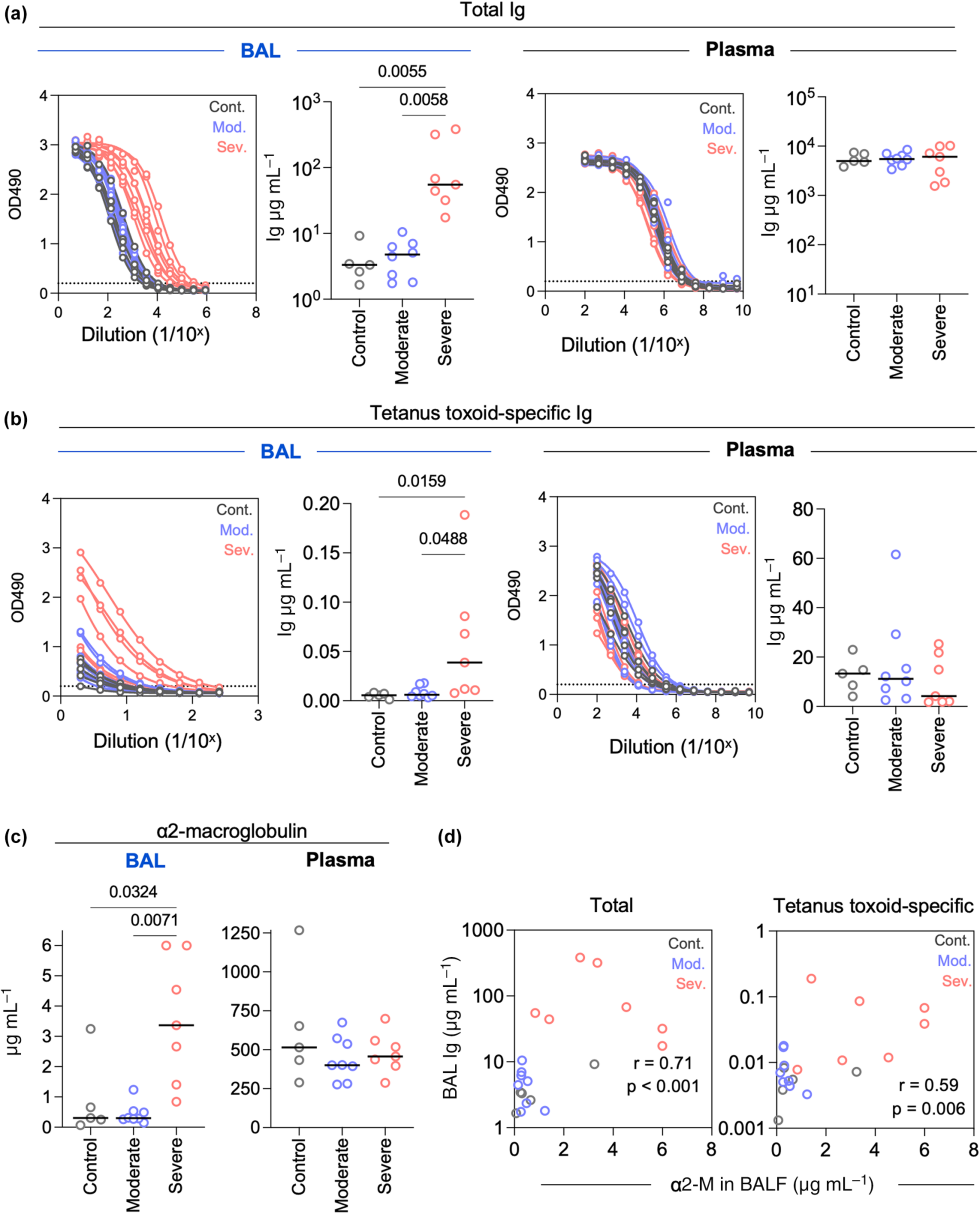
Increased exudation and plasma leakage in BAL samples from critically ill patients. **(a)** ELISA curves and interpolated concentrations of total Ig for BAL and plasma according to disease severity. **(b)** ELISA curves and interpolated concentrations of tetanus toxoid‐specific Ig for BAL and plasma according to disease severity. **(c)** Concentrations of α2‐macroglobulin in BAL and plasma according to disease severity. **(d)** Correlation between concentrations of α2‐macroglobulin in BAL and concentrations of total and tetanus toxoid‐specific Ig in BAL. In all panels of this figure, statistical significance was assessed by a Kruskal–Wallis test with Dunn's correction for multiple comparisons (*n* = 5 control subjects, *n* = 8 samples from 7 moderately ill subjects, *n* = 7 severely ill subjects).

We next assessed whether similar variability was present in BAL samples. We analysed paired BAL and plasma samples from five control subjects, seven moderately ill influenza A or B infected subjects and seven severely ill influenza B or SARS‐CoV‐2 infected subjects. One moderately ill influenza B infected subject provided two longitudinal samples, totalling a sample size of 20 for this analysis. Due to the limited volumes of sample available from some subjects, we analysed the total levels of antibody (2^o^ antibody against heavy and light chain), but not of specific isotypes. The total amount of antibodies detected varied considerably across subjects, ranging from 1.7 to 383.5 μg mL^−1^ total antibody (coefficient of variation 216.1%). This was in contrast to the more homogenous levels of antibodies in plasma from the same subjects (coefficient of variation 42.1%). We noted, however, that the variability could be attributed to the BAL samples from severely ill subjects (Figure 3a). Indeed, severely ill subjects had significantly higher levels of antibodies in the BAL, but not plasma, than moderately ill or control subjects (Figure 3a). Accordingly, we found that severely ill subjects had significantly higher levels of tetanus‐specific antibodies in the BAL, but not plasma, than moderately ill or control subjects (Figure 3b). When we focused the analysis on the 13 samples from control and moderately ill subjects, the total amount of antibodies detected only ranged from 1.7 to 8.8 μg mL^−1^ total antibody with a coefficient of variation of 63.6%.

We reasoned that severely ill subjects may have increased plasma exudation due to inflammation and/or vascular leakage in the airways because of damage to the epithelial cell barrier. To address this question, we measured the BAL and plasma levels of α2‐macroglobulin (α2‐M), which is considered a marker of plasma exudation.^12^, ^13^, ^14^ We found significantly higher levels of α2‐macroglobulin in severely ill subjects than in moderately ill or control subjects, which was not reflected in paired plasma samples (Figure 3c). The levels of α2‐M in BAL were positively correlated with the levels of total and tetanus‐specific Ig in the BAL (Spearman *r* = 0.71, *P* < 0.001 for total Ig; Spearman *r* = 0.59, *P* = 0.006 for tetanus) (Figure 3d). Overall, the antibodies present in the lower respiratory tract of severely ill subjects are likely confounded by increased plasma exudation and vascular leakage due to inflammation and tissue damage, and, thus, may not accurately reflect mucosal antibody responses.

## Discussion

Due to the increasing need to develop novel vaccine strategies that establish robust mucosal immunity in the airways, the assessment of antibodies in the upper and lower human respiratory tract is of great importance. Specifically, it is critical to understand (i) the relationship between the levels of mucosal antibodies and protection (from infection, disease and/or viral replication), (ii) the decay rates of antibodies in the respiratory mucosa and the cellular sources of long‐lived mucosal antibodies and (iii) how mucosal antibodies compare to circulating antibodies in terms of clonotypic composition and the resulting breadth of cross‐reactivity, potency of activity and extent of effector functions between the two compartments. Our study explored the utility and limitations of different samples that would be required to answer such questions in different populations of study participants (healthy, moderate disease and severe disease). Our study focused on the assessment of binding antibody titres, and further work is needed to establish robust assays that measure antibody‐mediated neutralisation in these samples. Nonetheless, binding antibody titres can be informative correlates of protection.^6^, ^7^, ^15^

The upper respiratory tract can be sampled by nasal lavage/aspiration; however, these methods are not performed routinely. As an alternative, saliva has been collected as a mucosal sample. While saliva can be collected readily and in minimally invasive ways, the extent to which it accurately reflects the nasal mucosa remains unclear. While our study does not address this question, we demonstrate that NP swabs can be used to readily sample the nasal mucosal surface, which may be a useful alternative, especially as they are routinely collected for diagnostic and surveillance purposes. Direct comparison between antibodies in saliva and NP swabs, as well as nasal aspirates, would be of interest as these may be preferable in certain settings. The detection of antibodies in diagnostic NP swabs in this study is consistent with other studies.^5^, ^8^ However, our analysis of 67 samples from patients with acute influenza exemplifies the potential variability in sampling that needs to be accounted for in quantitative analyses. This study evaluated the antibody content of nasopharyngeal swab samples and did not address any relationships between antibody content in this type of sample *versus* other sampling methods such as anterior‐ or mid‐turbinate nasal swab sampling. Further work is required to determine whether different types of swabs may perform better in terms of sampling by either providing a greater yield of antibodies and/or reduced variability. Highlighting the potential of NP swabs, recent analyses of antibodies in NP swabs have been instrumental in the correlating nasal antibodies to SARS‐CoV‐2 viral clearance^7^ as well as characterising the longevity of IgA antibodies in the human nasal mucosa after SARS‐CoV‐2 infection.^6^

Bronchoalveolar lavage is a common way of sampling the lower respiratory tract and the analysis of BAL samples following viral infection and/or vaccination is providing critical novel insights into important questions around mucosal immunity.^16^, ^17^, ^18^, ^19^ Frequently, however, such BAL samples may be excess material from bronchoscopy procedures performed on severely ill patients for clinical reasons. We highlight that such samples may be confounded by excessive plasma exudation and vascular leakage and may not accurately reflect mucosal immunity. Although these samples may be relatively easily accessible due to the logistical difficulties of obtaining BAL samples from healthy subjects, it is critical to appreciate their limitations and caution is urged in interpreting results. Measurement of markers like α2‐M could be useful in assessing the extent of plasma exudation prior to the analysis of such samples.

Due to the samples available for this study, we cannot determine how much of the variability in NP swab antibody levels is due to variability in sampling or true biological variability. Additional analyses are thus needed to determine the extent to which the observed variability is due to sampling or true biological variability between individuals. Finally, it would be important to determine correlations in the levels of antigen‐specific antibodies between the three compartments (serum, BAL, nasal mucosa), which we could not determine based on the samples of this study.

Overall, our findings provide important insights for the assessment of antibodies in the upper and lower respiratory tract of humans. Understanding antibody responses at these sites will be critical in developing vaccines that establish robust immunity at mucosal surfaces.

## Methods

### Participants and sample collection

We analysed paired plasma, NP swab and/or BAL samples that were obtained from influenza or SARS‐CoV‐2 infected subjects or control subjects (Supplementary table 1) as part of two previous studies.^11^, ^20^ Samples were selected for this study based on availability. Infection status (control, moderately ill, severely ill) was determined as previously described.^20^ Control subjects were healthy individuals who did not exhibit an influenza‐like illness for at least 60 days prior to outpatient bronchoscopy and BAL procedure. Moderately ill influenza subjects were individuals with symptomatic influenza A (IAV) or influenza B (IBV) virus infection that did not require hospitalisation prior to outpatient bronchoscopy and BAL procedure. Severely ill influenza subjects were individuals with severe IAV or IBV virus infection who were intubated for acute respiratory failure and BAL sampling was performed as a part of standard clinical care. Severely ill COVID‐19 subjects were individuals with severe COVID‐19 who were intubated for acute respiratory failure and BAL sampling was performed as part of standard clinical care.

For subjects with severe COVID‐19 or influenza, BAL fluid was obtained *via* bronchoscopy that was performed for a clinical reason, most frequently to evaluate for bacterial coinfection, at the discretion of the patient's treating physician. The BAL samples analysed in this study were excess material collected during that clinical bronchoscopy with BAL procedure. For severe influenza and severe COVID‐19 bronchoscopy procedures, the BAL was collected most frequently by wedging the bronchoscope within the right middle lobe bronchus. Each clinical procedure involved instilling 100 mL of sterile saline and collecting all returned fluid. Most of each sample was sent to the clinical laboratory for analysis, but ~10–15 mL of the BAL sample was provided to a member of the study.

For subjects with moderate illness and control subjects, samples were obtained during a scheduled elective outpatient research bronchoscopy. Briefly, following subject consent and safety screening with blood coagulation studies and a screening chest X‐ray, outpatient subjects received i.v. conscious sedation along with the application of lidocaine to the upper airway and vocal cords as the bronchoscope was passed into the airways. A brief visual inspection of the airways was performed to select an appropriate location for BAL collection; however, all outpatient research BAL samples were collected in the right middle lobe bronchus. BAL samples were collected by the instillation of 100–150 mL of sterile saline and collection of all returned lavage fluid.

Bronchoalveolar lavage fluid was processed by centrifugation at 400 *g* for 15 min at 4°C to pellet cells and debris. Clarified BAL fluid was stored at −80°C until further analysis. Nasopharyngeal swabs were collected using Becton Dickinson swabs (product number 220529, Becton Dickinson, New Jersey, USA) in 3 mL of universal virus transport media. NP swabs were collected by trained nurse clinical research coordinators using standard clinical methods. Following collection, NP swabs were vortexed vigorously for 1 min and aliquots were stored at −80°C until further analysis.

Informed consent was obtained from all subjects or their legally authorised representatives. The Institutional Review Board at Washington University in Saint Louis, USA, approved these studies (approval numbers 2017‐10‐220, 2018‐08‐115, 2019‐10‐011, 2020‐03‐085 and 2020‐06‐151). All studies complied with the ethical standards of the Helsinki Declaration.

### Assessment of antibodies by ELISA

ELISAs were performed in 96‐well plates (MaxiSorp; Thermo Fisher, MA, USA) which were coated with 100 μL of antigen diluted to 1 μg mL^−1^ in PBS, by incubating overnight at 4°C. The following antigens were used: tetanus toxoid (kindly provided by Daved Fremont), influenza virus H1 (A/Brisbane/2/2018; Sino Biological, Beijing, China), influenza virus H3 (A/Hong Kong/4801/2014; kindly provided by Florian Krammer), influenza virus B HA (B/Washington/2/2019; Sino Biological) or bovine serum albumin (negative control). To determine the total amount of antibody, plates were coated with AffiniPure Goat Anti‐Human IgA + IgG + IgM (H + L) (Jackson ImmunoResearch, PA, USA) at 1 μg mL^−1^ in PBS. Plates were blocked with 10% FBS and 0.05% Tween 20 in PBS. Plasma, NP swabs or BALF were serially diluted in blocking buffer and added to the plates. Plasma samples were diluted 1:50 and then 3‐fold serially for the analysis of antigen‐specific antibodies or 1:100 and the 5‐fold serially for the analysis of total antibody levels. NP swabs were diluted 1:3 and then 2‐fold serially for the analysis of antigen‐specific antibodies or 1:20 and the 5‐fold serially for the analysis of total antibody levels. BAL samples were diluted 1:2 and then 2‐fold serially for the analysis of antigen‐specific antibodies or 1:5 and the 3‐fold serially for the analysis of total antibody levels. Plates were incubated for 90 min at room temperature and then washed 3 times with 0.05% Tween 20 in PBS. Secondary antibodies conjugated to horseradish peroxidase were diluted in blocking buffer before adding to wells and incubating for 60 min at room temperature. The following secondary antibodies were used: goat anti‐human IgG (H + L)‐HRP (goat polyclonal against IgG heavy chain and Ig light chains, Jackson ImmunoResearch, 1:2500), goat anti‐human IgG (polyclonal, Fcγ fragment specific, 1:11 500, Jackson ImmunoResearch), goat anti‐human IgA (polyclonal, Jackson ImmuoResearch, 1:2500) and goat anti‐human IgG IgM (polyclonal, 1:4000, Caltag, ThermoFisher). Plates were washed 3 times with 0.05% Tween 20 in PBS and three times with PBS before the addition of o‐phenylenediamine dihydrochloride peroxidase substrate (Sigma‐Aldrich). Reactions were stopped by the addition of 1 M hydrochloric acid. Optical density measurements were recorded at 490 nm. For each experiment, purified IgG and IgA monoclonal antibodies produced in‐house as previously described^21^ or purified serum IgM (ChromPure Human IgM, Jackson ImmunoResearch) were used to generate standard curves from which concentrations were interpolated using a sigmoid 4PL curve in Graphpad Prism v9. Alternatively, endpoint titres were determined using 3× the average signal of background (2^o^ HRP antibody only) wells as a cut‐off by a sigmoid 4PL curve in Graphpad Prism v9.

### Assessment of α2‐macroglobulin by ELISA

The levels of α2‐macroglobulin were determined using Human Alpha 2‐M ELISA Kit (Human Alpha 2‐M ELISA Kit, Thermo Fisher) according to the manufacturer's instructions. Plasma samples were assayed at a 1:2000 dilution and BALF samples were assayed at a 1:2 dilution. All samples and standards were tested in duplicate. Concentrations were interpolated from a standard curve using a sigmoid 4PL curve in Graphpad Prism v9.

### Statistical analysis

Statistical significance was assessed by a Kruskal–Wallis test with Dunn's correction for multiple comparisons, a Friedman's test with Dunn's correction for multiple comparisons, a two‐way ANOVA with Sidak's correction for multiple comparisons, a Mann–Whitney test as detailed in the figure captions. Correlations were assessed using Pearson's correlation coefficient.

## Author contributions

**Marios Koutsakos:** Conceptualization; data curation; formal analysis; funding acquisition; investigation; methodology; supervision; visualization; writing – original draft; writing – review and editing. **Jackson S Turner:** Conceptualization; methodology; writing – review and editing. **M. Cristina Vazquez Guillamet:** Investigation; resources; writing – review and editing. **Daniel Reynolds:** Investigation; resources; writing – review and editing. **Tingting Lei:** Investigation; resources; writing – review and editing. **Derek E Byers:** Resources; writing – review and editing. **Ali H Ellebedy:** Conceptualization; funding acquisition; methodology; project administration; resources; supervision; writing – original draft; writing – review and editing. **Philip A Mudd:** Conceptualization; formal analysis; funding acquisition; investigation; methodology; project administration; resources; supervision; writing – original draft; writing – review and editing.

## Conflict of interest

The Ellebedy laboratory has received funding from Moderna, Emergent BioSolutions and AbbVie, which are unrelated to the data presented in the current study. JST has received consulting fees from Curevac. AHE has received consulting and speaking fees from InBios International, Inc, Fimbrion Therapeutics, RGAX, Mubadala Investment Company, Moderna, Pfizer, GSK, Danaher, Third Rock Ventures, Goldman Sachs and Morgan Stanley and is the founder of ImmuneBio Consulting. JST and AHE are recipients of a licensing agreement with Abbvie that is unrelated to the data presented in the current study. Other authors declare no conflicts of interest.

## Supporting information


Supplementary table 1
Supplementary figure 1

## Data Availability

The datasets generated during and/or analysed during the current study are available from the corresponding author on reasonable request.

## References

[1] Khoury DS , Cromer D , Reynaldi A *et al*. Neutralizing antibody levels are highly predictive of immune protection from symptomatic SARS‐CoV‐2 infection. Nat Med 2021; 27: 1205–1211. 34002089 10.1038/s41591-021-01377-8

[2] Sheikh‐Mohamed S , Sanders EC , Gommerman JL , Tal MC . Guardians of the oral and nasopharyngeal galaxy: IgA and protection against SARS‐CoV‐2 infection. Immunol Rev 2022; 309: 75–85. 35815463 10.1111/imr.13118 PMC9349649

[3] Gould VMW , Francis JN , Anderson KJ , Georges B , Cope AV , Tregoning JS . Nasal IgA provides protection against human influenza challenge in volunteers with low serum influenza antibody titre. Front Microbiol 2017; 8: 900. 28567036 10.3389/fmicb.2017.00900 PMC5434144

[4] Ravichandran S , Grubbs G , Tang J *et al*. Systemic and mucosal immune profiling in asymptomatic and symptomatic SARS‐CoV‐2‐infected individuals reveal unlinked immune signatures. Sci Adv 2021; 7: eabi6533. 34644111 10.1126/sciadv.abi6533 PMC8514093

[5] Planas D , Bruel T , Grzelak L *et al*. Sensitivity of infectious SARS‐CoV‐2 B.1.1.7 and B.1.351 variants to neutralizing antibodies. Nat Med 2021; 27: 917–924. 33772244 10.1038/s41591-021-01318-5

[6] Marking U , Bladh O , Havervall S *et al*. 7‐month duration of SARS‐CoV‐2 mucosal immunoglobulin‐a responses and protection. Lancet Infect Dis 2023; 23: 150–152. 36640796 10.1016/S1473-3099(22)00834-9 PMC9833832

[7] Havervall S , Marking U , Svensson J *et al*. Anti‐spike mucosal IgA protection against SARS‐CoV‐2 omicron infection. N Engl J Med 2022; 387: 1333–1336. 36103621 10.1056/NEJMc2209651 PMC9511632

[8] Crescenzo‐Chaigne B , Behillil S , Enouf V *et al*. Nasopharyngeal and serological anti SARS‐CoV‐2 IgG/IgA responses in COVID‐19 patients. J Clin Virol Plus 2021; 1: 100041. 35262023 10.1016/j.jcvp.2021.100041 PMC8474802

[9] Gonzalez‐Quintela A , Alende R , Gude F *et al*. Serum levels of immunoglobulins (IgG, IgA, IgM) in a general adult population and their relationship with alcohol consumption, smoking and common metabolic abnormalities. Clin Exp Immunol 2008; 151: 42–50. 18005364 10.1111/j.1365-2249.2007.03545.x PMC2276914

[10] Peebles RS Jr , Liu MC , Lichtenstein LM , Hamilton RG . IgA, IgG and IgM quantification in bronchoalveolar lavage fluids from allergic rhinitics, allergic asthmatics, and normal subjects by monoclonal antibody‐based immunoenzymetric assays. J Immunol Methods 1995; 179: 77–86. 7868927 10.1016/0022-1759(94)00275-2

[11] Turner JS , Lei T , Schmitz AJ *et al*. Impaired cellular immune responses during the first week of severe acute influenza infection. J Infect Dis 2020; 222: 1235–1244. 32369589 10.1093/infdis/jiaa226 PMC7768688

[12] Persson C . Early humoral defence: contributing to confining COVID‐19 to conducting airways? Scand J Immunol 2021; 93: e13024. 33523532 10.1111/sji.13024 PMC7994976

[13] Nocker RE , Weller FR , Out TA , de Riemer MJ , Jansen HM , van der Zee JS . A double‐blind study on the effect of inhaled corticosteroids on plasma protein exudation in asthma. Am J Respir Crit Care Med 1999; 159: 1499–1505. 10228117 10.1164/ajrccm.159.5.9806116

[14] Ratjen F , Havers W , Braun J . Intrapulmonary protein leakage in immunocompromised children and adults with pneumonia. Thorax 1999; 54: 432–436. 10212109 10.1136/thx.54.5.432 PMC1763773

[15] Ng S , Nachbagauer R , Balmaseda A *et al*. Novel correlates of protection against pandemic H1N1 influenza a virus infection. Nat Med 2019; 25: 962–967. 31160818 10.1038/s41591-019-0463-x PMC6608747

[16] Tang J , Zeng C , Cox TM *et al*. Respiratory mucosal immunity against SARS‐CoV‐2 after mRNA vaccination. Sci Immunol 2022; 7: eadd4853. 35857583 10.1126/sciimmunol.add4853 PMC9348751

[17] Mitsi E , Diniz MO , Reiné J *et al*. Long‐term respiratory mucosal immune memory to SARS‐CoV‐2 after infection and vaccination. bioRxiv 2023: 2023.2001.2025.525485. 10.1038/s41467-023-42433-w PMC10603102 37884506

[18] Sterlin D , Mathian A , Miyara M *et al*. IgA dominates the early neutralizing antibody response to SARS‐CoV‐2. Sci Transl Med 2021; 13: abd2223. 10.1126/scitranslmed.abd2223 PMC7857408 33288662

[19] Ruiz MJ , Siracusano G , Cottignies‐Calamarte A *et al*. Persistent but dysfunctional mucosal SARS‐CoV‐2‐specific IgA and low lung IL‐1β associate with COVID‐19 fatal outcome: a cross‐sectional analysis. Front Immunol 2022; 13: 842468. 36248831 10.3389/fimmu.2022.842468 PMC9560774

[20] Reynolds D , Vazquez Guillamet C , Day A *et al*. Comprehensive immunologic evaluation of bronchoalveolar lavage samples from human patients with moderate and severe seasonal influenza and severe COVID‐19. J Immunol 2021; 207: 1229–1238. 34348975 10.4049/jimmunol.2100294 PMC8387368

[21] Turner JS , Zhou JQ , Han J *et al*. Human germinal centres engage memory and naive B cells after influenza vaccination. Nature 2020; 586: 127–132. 32866963 10.1038/s41586-020-2711-0 PMC7566073

