# Supplementary material for: Assessment of antibodies in the upper and lower human respiratory tract at steady state and after respiratory viral infection
Source: Clin Transl Immunology. 2023 Aug 8;12(8):e1460. doi: 10.1002/cti2.1460 (PMC10410120; doi:10.1002/cti2.1460)
Supplement: Supplementary file 1 — Supplementary table 1 Supplementary figure 1 [file CTI2-12-e1460-s001.docx]

**Supplementary Material**

**Supplementary table 1. Cohort and sample details**

| **Patient ID** | **Infection status** | **Severity group** | **Age** | **Sex** | **Days post-onset** | **Plasma available** | **NP swab available** | **BAL available** |
| --- | --- | --- | --- | --- | --- | --- | --- | --- |
| 1718001 | IAV | moderate | 39 | M | 2 | Y | Y | N |
| 1718002 | IAV | severe | 35 | F | 4 | Y | Y | N |
| 1718003 | IAV | moderate | 34 | F | 5 | Y | Y | N |
| 1718004 | IAV | moderate | 23 | F | 5 | Y | Y | N |
| 1718006 | IAV | moderate | 34 | F | 7 | Y | Y | N |
| 1718007 | IAV | severe | 64 | M | 5 | Y | Y | N |
| 1718008 | IAV | moderate | 28 | M | 4 | Y | Y | N |
| 1718009 | IAV | moderate | 61 | F | 3 | Y | Y | N |
| 1718010 | IAV | moderate | 27 | F | 8 | Y | Y | N |
| 1718011 | IAV | severe | 61 | M | 14 | Y | Y | N |
| 1718012 | IAV | moderate | 67 | F | 1 | Y | Y | N |
| 1718013 | IBV | severe | 58 | M | 4 | Y | Y | N |
| 1718014 | IBV | severe | 60 | M | 4 | Y | Y | N |
| 1718015 | IAV | severe | 52 | F | 1 | Y | Y | N |
| 1718016 | IAV | severe | 69 | F | 2 | Y | Y | N |
| 1718017 | IAV | moderate | 57 | M | 1 | Y | Y | N |
| 1718018 | IAV | moderate | 38 | F | 1 | Y | Y | N |
| 1718019 | IBV | moderate | 73 | M | 2 | Y | Y | N |
| 1718020 | IAV | moderate | 74 | F | 3 | Y | Y | N |
| 1718022 | IAV | severe | 77 | M | 3 | Y | Y | N |
| 1718024 | IAV | moderate | 60 | M | 1 | Y | Y | N |
| 1718025 | IBV | moderate | 51 | M | 4 | Y | Y | N |
| 1718026 | IAV | severe | 62 | F | 7 | Y | Y | N |
| 1718027 | IAV | moderate | 61 | M | 1 | Y | Y | N |
| 1718028 | IAV | severe | 48 | M | 3 | Y | Y | N |
| 1819001 | IAV | moderate | 36 | F | 2 | Y | Y | N |
| 1819003 | IAV | moderate | 18 | M | 3 | Y | Y | N |
| 1819004 | IAV | moderate | 65 | F | 13 | Y | Y | N |
| 1819008 | IAV | moderate | 57 | M | 7 | Y | Y | N |
| 1819009 | IAV | moderate | 18 | F | 2 | Y | Y | N |
| 1819013 | IAV | moderate | 25 | F | 1 | Y | Y | N |
| 1819014 | IAV | severe | 73 | F | 1 | Y | Y | N |
| 1819022 | IAV | moderate | 26 | F | 3 | Y | Y | N |
| 1819027 | IAV | moderate | 39 | M | 2 | Y | Y | N |
| 1819028 | IAV | severe | 71 | F | 4 | Y | Y | N |
| 1819030 | IAV | severe | 72 | M | 3 | Y | Y | N |
| 1819035 | IAV | moderate | 59 | M | 1 | Y | Y | N |
| 1819037 | IAV | moderate | 20 | F | 2 | Y | Y | N |
| 1819039 | IAV | moderate | 32 | F | 4 | Y | Y | N |
| 1819041 | IAV | severe | 55 | F | 7 | Y | Y | N |
| 1819042 | IAV | moderate | 78 | F | 3 | Y | Y | N |
| 1819043 | IAV | severe | 57 | F | 2 | Y | Y | N |
| 1819044 | IAV | severe | 52 | M | 1 | Y | Y | N |
| 1920001 | IBV | moderate | 18 | F | 2 | Y | Y | N |
| 1920002 | IBV | moderate | 27 | F | 7 | Y | Y | N |
| 1920003 | IAV | severe | 76 | M | 4 | Y | Y | N |
| 1920004 | IBV | moderate | 32 | F | 1 | Y | Y | N |
| 1920006 | IAV | moderate | 89 | F | 3 | Y | Y | N |
| 1920007 | IAV | moderate | 65 | F | 3 | Y | Y | N |
| 1920008 | IAV | severe | 51 | F | 2 | Y | Y | N |
| 1920009 | IAV | moderate | 30 | F | 1 | Y | Y | N |
| 1920010 | IBV | moderate | 26 | M | 1 | Y | Y | N |
| 1920011 | IAV | moderate | 33 | F | 6 | Y | Y | N |
| 1920012 | IBV | severe | 43 | F | 4 | Y | Y | N |
| 1920013 | IAV | severe | 54 | M | 7 | Y | Y | N |
| 1920014 | IAV | moderate | 58 | M | 7 | Y | Y | N |
| 1920015 | IBV | moderate | 34 | M | 2 | Y | Y | N |
| 1920016 | IAV | severe | 49 | M | 4 | Y | Y | N |
| 1920019 | IAV | severe | 50 | M | 5 | Y | Y | N |
| 1819B004 | IAV | moderate | 40 | F | 18 | Y | N | Y |
| 1819B007 | IAV | moderate | 27 | M | 9 | Y | N | Y |
| 1819B008 | IBV | moderate | 28 | M | 3 | Y | Y | N |
| 1920B004 | IBV | moderate | 29 | F | 4 | Y | Y | Y* |
| 1920B005 | IBV | moderate | 23 | F | 6 | Y | Y | Y |
| 1920B007 | IBV | moderate | 29 | F | 3 | Y | Y | Y |
| 1920B008 | IAV | moderate | 31 | M | 3 | Y | Y | Y |
| 1920B009 | IAV | severe | 45 | M | 1 | Y | Y | N |
| 1920B011 | IBV | severe | 26 | F | 7 | Y | Y | Y |
| 1920B013 | IBV | moderate | 41 | M | 6 | Y | Y | Y |
| 350-428 | COVID-19 | severe | 59 | F | 11 | Y | N | Y |
| 350-441 | COVID-19 | severe | 79 | M | 11 | Y | N | Y |
| C11_14 | COVID-19 | severe | 60 | F | 14 | Y | N | Y |
| C12_11 | COVID-19 | severe | 53 | M | 11 | Y | N | Y |
| C8 | COVID-19 | severe | 63 | F | 5 | Y | N | Y |
| C9_32 | COVID-19 | severe | 72 | F | 32 | Y | N | Y |
| 1819B001 | Control | n/a | 28 | F | n/a | Y | Y | Y |
| 1920B002 | Control | n/a | 29 | M | n/a | Y | Y | Y |
| 2223B001 | Control | n/a | 31 | M | n/a | Y | Y | Y |
| 2223B002 | Control | n/a | 31 | F | n/a | Y | Y | Y |
| 2223B003 | Control | n/a | 33 | M | n/a | Y | Y | Y |
| 2223B004 | Control | n/a | 23 | M | n/a | Y | Y | Y |
| 2223B005 | Control | n/a | 23 | M | n/a | Y | Y | Y |
| 2223B008 | Control | n/a | 23 | F | n/a | Y | Y | Y |
| M: male; F: female; *2 longitudinal samples available | | | | | | | | |

**Supplementary figure 1. Analysis of antibodies in BAL samples and nasopharyngeal swabs.** (a) Endpoint titres of each isotype across sample types in paired samples from control subjects (n=8). (b) Standard curves used for each isotype to interpolate concentrations (μg ml^−1^). (c) Correlation between endpoint titres and interpolated concentrations of antibodies in plasma, NP swab and BAL samples from control subjects (n=72 datapoints, pooled subjects, isotypes and sample type). (d) Concentrations of total Ig and IgA in serum from EDFLU subjects grouped based on disease severity (moderate n=43, severe n=24) and control subjects (n=6). Statistical significance was assessed by a Kruskal-Wallis test with Dunn’s correction for multiple comparisons. (e) Correlation between total and HA-specific antibodies in NP swabs.
